# Diverse Routes of Allograft Tolerance Disruption by Memory T Cells

**DOI:** 10.3389/fimmu.2020.580483

**Published:** 2020-10-08

**Authors:** Ronald G. Gill, Adam L. Burrack

**Affiliations:** ^1^ Departments of Surgery and Immunology and Microbiology, University of Colorado Denver, Aurora, CO, United States; ^2^ Department of Microbiology and Immunology, University of Minnesota Medical School, Minneapolis, MN, United States

**Keywords:** immune memory, autoimmunity, tolerance, transplantation, infection, vaccination

## Abstract

Memory T lymphocytes constitute a significant problem in tissue and organ transplantation due their contribution to early rejection and their relative resistance to tolerance-promoting therapies. Memory cells generated by environmental antigen exposure, as with T cells in general, harbor a high frequency of T cell receptors (TCR) spontaneously cross-reacting with allogeneic major histocompatibility complex (MHC) molecules. This phenomenon, known as ‘heterologous’ immunity, is thought to be a key barrier to transplant tolerance induction since such memory cells can potentially react directly with essentially any prospective allograft. In this review, we describe two additional concepts that expand this commonly held view of how memory cells contribute to transplant immunity and tolerance disruption. Firstly, autoimmunity is an additional response that can comprise an endogenously generated form of heterologous alloimmunity. However, unlike heterologous immunity generated as a byproduct of indiscriminate antigen sensitization, autoimmunity can generate T cells that have the unusual potential to interact with the graft either through the recognition of graft-bearing autoantigens or by their cross-reactive (heterologous) alloimmune specificity to MHC molecules. Moreover, we describe an additional pathway, independent of significant heterologous immunity, whereby immune memory to vaccine- or pathogen-induced antigens also may impair tolerance induction. This latter form of immune recognition indirectly disrupts tolerance by the licensing of naïve alloreactive T cells by vaccine/pathogen directed memory cells recognizing the same antigen-presenting cell *in vivo.* Thus, there appear to be recognition pathways beyond typical heterologous immunity through which memory T cells can directly or indirectly impact allograft immunity and tolerance.

## Introduction

Memory T cells constitute a formidable obstacle both for preventing early graft rejection and for the eventual induction of allograft tolerance. For example, memory CD8 T cells can trigger early aggressive rejection of cardiac allograft rejection in mice (1). Importantly, memory T cells are also relatively resistant to tolerance-promoting therapies (2–4). An important property of memory cells thought to be especially relevant for impairing allograft survival is their strong ‘heterologous’ reactivity to allogeneic MHC molecules. The concept of heterologous immunity originated by the observation that humoral or cellular immunity to one pathogen could impart reactivity to a secondary, unrelated pathogen (5). This property is found in memory T cells generated in response to one virus that that can cross-react with a new unrelated viral infection (6, 7). This term has been borrowed by the transplantation field to describe a specific feature of memory T cells that imparts a high degree of cross-reactivity to allogeneic MHC molecules (8, 9). This phenomenon is almost certainly due to the high intrinsic bias of the TCR repertoire for MHC recognition (10, 11). Thus, simply by chance, any polyclonal antigen-specific T cell response would be expected to contain a significant subpopulation of allogeneic MHC-reactive T cells. This concept is strikingly illustrated by the findings from Amir et al. showing that nearly half of CD4 and CD8 virus-specific human T cell clones cross-reacted to at least one allogeneic HLA allele (12). A high degree of cross-reactivity to alloantigens by pathogen-induced T cells has also been demonstrated in mouse infection models (13–15).

## Autoimmunity as an Endogenous Source of Heterologous Allograft Immunity

There has been interest in the transplant field for how alloimmunity may initiate nascent autoimmunity that can impact the graft. This has been especially evident in chronic allograft reactivity in lung transplantation (16, 17). However, the converse may also be true; pre-existing autoimmunity may be a source of potential alloimmunity in the form of heterologous immunity. We have had a long-standing interest in islet transplantation using the non-obese diabetic (NOD) model of spontaneous autoimmune Type 1 diabetes. NOD mice have a multi-factorial predisposition for developing diabetes due to T and B cell dependent islet beta cell-specific autoimmunity (18–20). Importantly, diseased NOD mice destroy syngeneic (NOD) pancreas (21) or isolated islet (22) transplants through a process of recurrent disease, a phenomenon that also occured in non-immunosuppressed Type 1 patients receiving a partial pancreas transplant from a non-diseased identical twin (23). Moreover, NOD mice also show a strong response to islet allografts (22, 24, 25). As such, the NOD mouse model is highly useful for studying islet transplant autoimmunity and alloimmunity, including potential heterologous immunity, in the setting of Type 1 diabetes.

Based on the discussion above, the autoimmune T cell repertoire, like any polyclonal T cell population, would be expected to have a high degree of cross-reactivity to allogeneic MHC molecules. Interestingly, a survey of established autoreactive (islet antigen-specific) T cell clones derived from NOD mice revealed that over one third cross-reacted to one or more of three allogeneic MHC haplotypes (26), a result conceptually similar to what had been found previously for human virus-specific T cell clones (12). Based on this concept, we interrogated the TCR specificity of T cells infiltrating MHC-unrelated islet allografts grafted into spontaneously diabetic NOD mice. Consistent with results from screening autoreactive T cell clones, TCRs from islet allografts were profoundly enriched with dual autoreactive/alloreactive specificities (26). Thus, autoimmunity can be a source *endogenously* generated heterologous immunity contributing to allograft rejection.

### Heterologous Autoreactive T Cells With Alloreactivity: One or Two TCRs?

While the simultaneous reactivity of individual T cells for both self-MHC-restricted cognate antigens and allogeneic MHC molecules has been apparent for many years (27), it is not always clear whether this is due to a single TCR α/β pair or due to two separate TCRs on a given cell. There is ample reason to posit that autoreactive T cells demonstrating additional alloreactivity could be due to the contribution of two separate TCRs. A significant percentage (estimated to be roughly between 1-8%) of mature mouse (28–30) and human (31) peripheral T cells express two TCRs, presumably due to a substantial frequency of developing T cells expressing two functional TCR α chains (32). Moreover, dual TCR-expressing T cells indeed have a high frequency of an alloreactive second receptor (33, 34), and these can play an important role in triggering graft-versus-host disease in mice (33). It is conceivable, then, that autoreactive T cells demonstrating cross-reactive alloreactivity could be the result of two separate TCR specificities on the same cells.

Conversely, when studying a dual TCR-expressing T cell clone with both self-MHC-restricted peptide specify (OVA) *and* alloreactvity, Malissen et al. found that only one the two TCR α/β pairs imparted this dual reactivity (35). This demonstrates that a single TCR can possess combined nominal antigen plus cross-reactive allo-specificity. This concept was supported by studies involving high-throughput sequencing of a large repertoire of TCR transcripts from T cells targeting islet allografts in spontaneously diabetic NOD mice (26). Importantly, screening the antigen reactivity of highly expressed TCRs indicated that single TCR α/β pairs conferred simultaneous dual autoantigen/alloantigen (MHC) reactivity (26). Thus, the predominant heterologous immunity identified by this approach could be accounted for by single autoreactive TCRs with clear cross-reactivity to allogeneic MHC molecules. Of course, these finds do not preclude the potential of heterologous alloimmunity emerging from autoreactive T cells being the result of a second TCR. However, results to date suggest that the most frequent source of simultaneous autoreactive/alloreactive T cells in islet transplants in the setting of autoimmunity is the result of a single, cross-reactive TCR.

### Conventional Antigen-Stimulated Versus Autoimmune Heterologous Immunity: A ‘Trojan Horse’ Model of Allograft Immunity

One potentially key difference between memory T cells generated by past antigen challenge and ongoing autoimmunity may simply be in the activation state of antigen-experienced T cells in these two scenarios. In fact, memory T cells may not be completely resistant to tolerance induction (2). For example, naïve mice can be tolerized to tissue and organ transplants despite bearing a degree of memory T cells generated by environmental antigen exposure. If this is the case, then the impact of immune memory on allograft rejection and tolerance may be related in part to the pre-transplant burden of pre-existing alloreactive T cell memory (8, 36). However, the activation state of memory cells may also impact their potential to be tolerized. In most cases, one would expect memory cells from past antigen exposure to be in a more quiescent state of central memory (2). However, the autoimmune T cell pool may be experiencing persistent activation/re-activation in the host, including those cells expressing cross-reactive alloimmunity. This means that the alloimmune component found in autoimmune disease may already be in a heightened activation state and potentially more challenging to tolerize. While NOD mice have a variety of tolerance defects (37, 38) the presence of alloreactvity found within the smoldering autoreactive repertoire may contribute to the dramatic resistance of NOD mice to allograft tolerance, even toward tissues/organs for which they have no apparent autoimmunity (37). It will be most interesting to test this concept in future studies.

**Figure 1:**
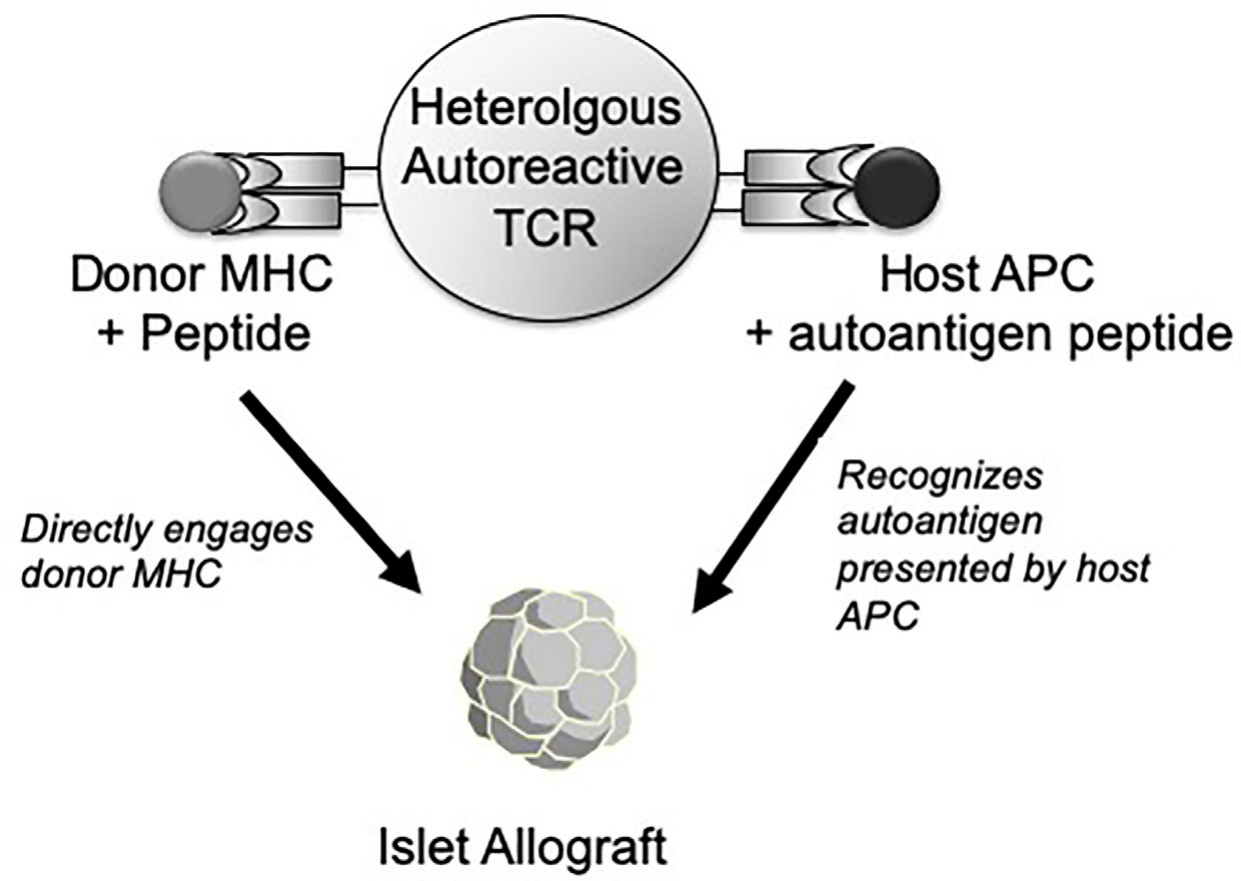
Depiction of an autoreactive (islet-specific) T cell with a TCR with both autoantigen specificity and cross-reactivity to allogenic MHC molecules. In response to an islet allograft, this type of heterologous TCR can recognize a host MHC-restricted, graft-derived autoantigen peptide presented by host APC (right side). Alternatively, the same TCR may also directly recognize a native donor MHC molecule plus an unidentified peptide expressed by the graft (left side). As such, the same T cell has the potential to interact with the islet graft through either a host MHC-restricted (autoreactive) or donor MHC-restricted (alloreactive) recognition pathway.

In addition, there is a second and more unusual property of heterologous (alloreactive) autoreactive T cells that may make them especially virulent as mediators of islet rejection. In the conventional view of heterologous immunity, antigen-experienced memory cells contribute to allograft immunity and tolerance resistance due to their chance cross-reactivity to the graft, unrelated to the specificity of the original stimulating antigen. However, in the case of autoimmunity, heterologous T cells have the potential to interact with graft through two qualitatively distinct recognition pathways simultaneously (**Figure 1**). One route of islet graft interaction can be through the recognition of self MHC-restricted islet autoantigens acquired from the transplant and processed and presented by host antigen-presenting cells (APCs). We previously found that monoclonal BDC2.5 TCR transgenic CD4 T cells without allogeneic cross-reactivity could nevertheless recognize allograft-derived autoantigens processed by host APCs and destroy islet allografts though this type of indirect autoantigen recognition (39). Thus, this autoreactive specificity alone was sufficient to trigger allograft rejection. However, since polyclonal autoreactive T cells targeting the islet graft also contain cross-reactive, alloreactive T cells (26), some of these cells can *also* directly recognize the native allogeneic MHC expressed by the graft. An example of this phenomenon is a CD4 TCR (9860-A3B3) isolated from an MHC mismatched C3H (H-2^k^) islet graft in NOD mice. This TCR recognizes an islet-associated Chromogranin A peptide presented by the NOD MHC class II I-A^g7^ while *also* directly recognizing allogeneic I-A^k^ expressed by the donor (26). Thus, this unusual situation could represent a sort of ‘Trojan Horse’ phenomenon in the islet graft in which the influx of T cells responding to autoantigens also ferries in a cohort of heterologous alloreactive T cells that can directly engage the allograft MHC. This simultaneous graft recognition through either auto- or allo- specificities could account for the accelerated response to MHC unrelated islet allografts in NOD mice despite the lack of intentional prior alloantigen exposure in these mice (24, 25). This property of heterologous immunity within autoimmune T cells could potentially be a general dilemma in controlling allograft rejection in the setting of autoimmune disease.

## An Additional and Less Apparent Route of Tolerance Blockade by Memory T Cells

A major ongoing goal in transplantation is to transition from chronic non-specific immunosuppression towards the induction of antigen (donor)-specific tolerance. To this point, this discussion has centered on heterologous immunity by memory T cells to allogeneic MHC molecules themselves (either from prior antigen exposure or *via* autoimmunity) as a key problem in transplant immunity and tolerance. As such, the importance of assessing pre-existing humoral or cellular immunity to donor MHC has been a major focus of screening efforts in transplantation (40–42). While such efforts are clearly warranted, there are potentially alternative routes whereby T cell memory could impair tolerance induction without a requirement for substantial heterologous immunity to the donor MHC (43). Unfortunately, the metagenome of both organ donors and recipients encode a variety of non-self antigens, such as those derived from microbiota (44, 45) or from latent infections such as CMV and EBV (46–49) that are clearly associated with impaired allograft outcomes in clinical transplantation. It is clear that the activation of anti-viral immunity can abrogate allograft tolerance (50, 51), possibly by the induction of inflammation that itself may non-specifically impair tolerance induction (52, 53).

One could assume that much of the impairment of tolerance induced by recipient responses to donor-associated pathogens is related to either heterologous immunity generated during the pathogen response and/or to the associated inflammation. We propose another more provocative form of host immunity to donor-derived non-MHC antigens that also could impair tolerance induction. This problem of donor-derived, non-self, non-MHC antigens has arguably been under-represented in most small animal studies. This being the case, we developed a model system in which the donor expressed a non-self transgenic antigen (OVA) to which the host was immune *via* vaccination (43), a scenario that could have relevance to clinical transplantation in which vaccination might protect from a donor-derived pathogen (54). Tolerance was induced using a common approach of administering a pre-transplant donor-specific transfusion (DST) in the form of donor spleen cells plus costimulation blockade (55, 56). Interestingly, host anti-OVA vaccination alone was innocuous, generating negligible anti-donor heterologous alloimmunity and had no impact on tolerance induction to wild-type allografts. Even peri-transplant re-activation of host anti-OVA reactivity did not impair tolerance induction. However, treatment with an OVA-expressing allogeneic DST in an OVA-immune recipient profoundly abrogated tolerance induction, even if the subsequent allograft did not express OVA (43).

## Tolerance Disruption of Naïve T Cells by Memory T Cells *via* Linked Antigen Presentation

**Figure 2:**
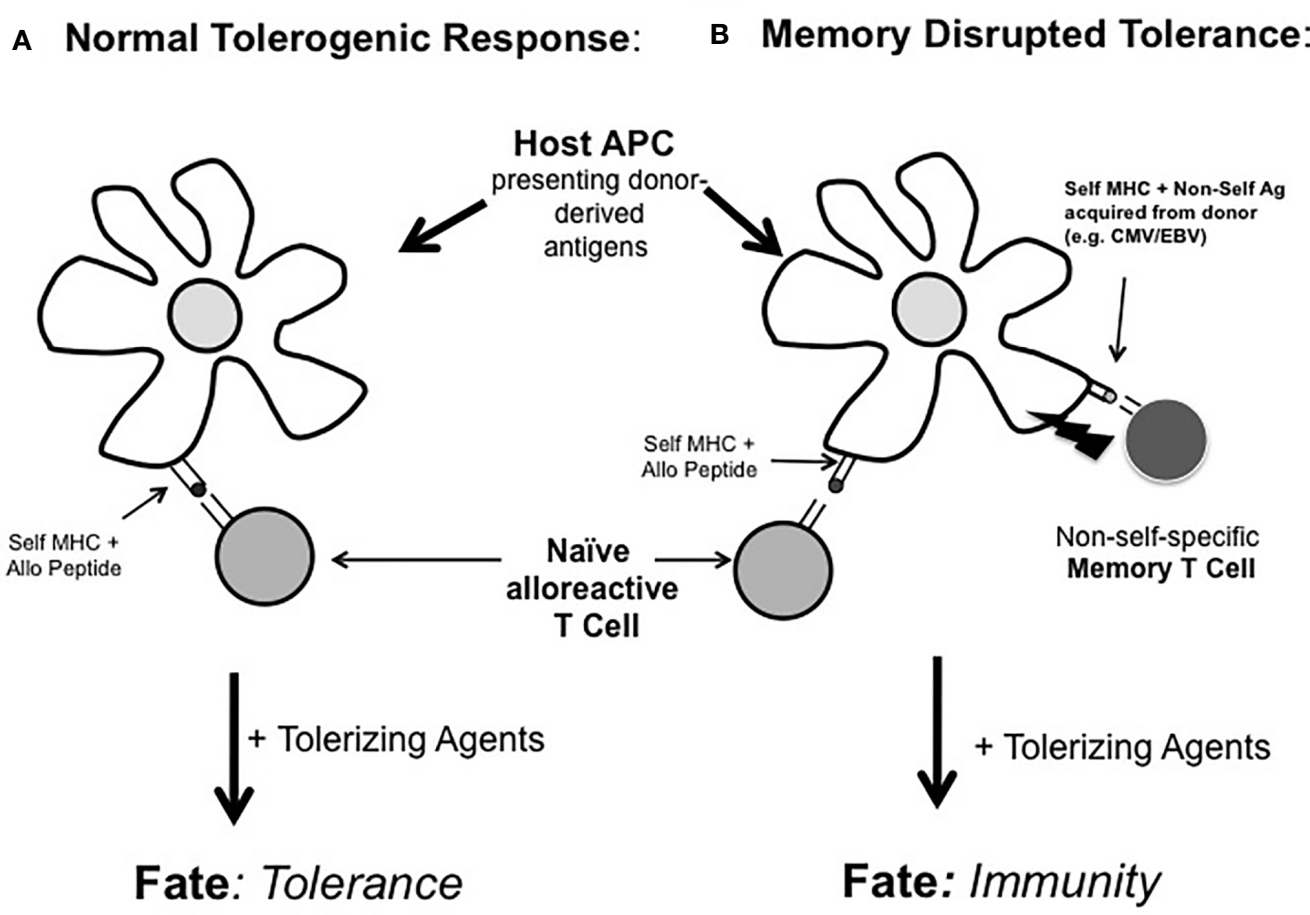
Working model of tolerance blockade by linked recognition of alloantigens and non-MHC donor antigens. In **(A)** host naïve T cells responding to donor antigens acquired by host APCs are amendable to tolerance induction by tolerance-promoting agents. In **(B)**, if these same APCs also acquire other donor-derived antigens to which the host has pre-existing immunity (e.g., to non-self pathogen-derived antigens or autoantigens), tolerance is disrupted at the level of the APC. In this case, the fate of such uncommitted alloreactive T cells is diverted from tolerance to immunity.

A key feature of this admittedly contrived system was that that the alloantigen and non-self (OVA) antigen had to be presented on the same APC in order to disrupt tolerance (**Figure 2**). That is, ‘linked’ recognition of the vaccine-directed antigen and the alloantigen was required for tolerance blockade (43). This scenario illustrates the potential for an alternative route whereby memory cells may impact the microenvironment during initial tolerance induction at the level of antigen presentation, not *via* donor MHC recognition, but rather through the recognition of another non-MHC antigen introduced by donor cells. Currently, probably the most recognizable concept involving T cells influencing one another *via* recognition of the same APC is that of ‘linked suppression’ in which putative regulatory T cells inhibits the function of another uncommitted T cell through interacting with the same APC (57). However, the concept of linked recognition leading to cell *activation* is actually considerably older. The original description of ‘linked’ antigen recognition referred to the observation of the carrier-hapten phenomenon in which the ‘helper’ determinant for antigen formation required physical linkage between the ‘helper’ determinant and the antibody specificity (58). This concept was later adapted to refer to the finding that helper T cells for the generation of cytotoxic T cells required recognition of the same APC *in vivo* (59). Three seminal studies later found that the basis of such CD4 T cell help for CD8 T cells was in the form of CD40:CD154 interactions with the APC resulting in the licensing of such APCs to activate other T cells (60–62). We had proposed that such T-T cell collaboration could be bi-directional in that CD4 and CD8 T cells could potentially influence one another through linked recognition of the APC (63). This latter concept could explain how memory CD8 T cells could disrupt T cell tolerance and promote allograft rejection instead (64). Of course, while this model of tolerance disruption required memory CD8 T cells (43), there is clear evidence that both CD4 (65–67) and CD8 (64, 65, 68) T cells can be involved in tolerance blockade in pre-clinical models. However, it is usually unclear how specific memory T cell subsets actually impair tolerance induction.

In what situation might this type of memory cell reactivity be important in transplantation? In the setting of autoimmunity or c donor pathogen infections such as CMV and EBV (46–49), the host could be immune to donor-derived, non-MHC antigens without obvious pre-transplant anti-donor MHC immunity. However, depending on the tissue distribution of autoantigens or donor pathogen-derived antigens, memory cells for these antigens could disrupt tolerance induction by diverting the naïve T cells recognizing the same APC from a tolerized fate to an effector phenotype (**Figure 2**). Because the existing host T cell memory to donor-derived, non-self antigens was self-MHC restricted in this linked recognition model (65), we would propose that the ‘indirect’ pathway of alloantigen recognition by host APCs was chiefly involved in disrupting tolerance. This could contrast sharply with how heterologous memory T cells (i.e., T cells with direct donor MHC reactivity) disrupt tolerance. Such donor MHC cross-reactive T cells may influence tolerance through the ‘direct’ pathway of antigen recognition. Future studies are needed to define the specific cellular interactions required by memory T cells to impair tolerance. Moreover, this tolerance blockade could occur without significant evidence of conventional heterologous anti-donor MHC reactivity. If this alternate and less apparent route of memory T cell tolerance blockade is significant, it implies that assessing pre-transplant anti-donor MHC reactivity alone may not be sufficient to predict the potential success of tolerance-promoting therapies. It may also be important to more carefully assess the presence of donor-derived pathogens and the corresponding host immunity to these antigens or to autoantigens.

## Concluding Remarks

The role of memory T cells for providing resistance to allograft tolerance induction is well established. Moreover, the high degree of heterologous anti-donor MHC alloreactivity found within memory T cell populations is rightly considered a major potential source of tolerance disruption. By this view, the implied paradigm is that memory cells behave essentially as directly allo-sensitized cells that are resistant to regulation. However, there are other routes of memory cell specificity that expand and perhaps complicate this straightforward view (summarized in **Table 1**). For example, autoimmunity may constitute a form of ongoing memory T cell generation and heterologous alloreactivity that does not require exogenous antigen exposure. Also, heterologous autoreactive T cells have the unusual potential for recognizing autoantigen-expressing allografts through autoreactive and alloreactive specificities simultaneously. Alternatively, memory T cells can potentially subvert tolerance induction by recognizing donor-derived, non-MHC antigens (such as autoantigens or from pathogens) co-presented on APCs with conventional alloantigens resulting in the disruption of tolerance by naïve alloreactive T cells. Importantly, this latter form of antigen recognition could impair tolerance even in the absence of significant anti-donor MHC reactivity. The relative significance of this latter route of tolerance blockade by memory T cells requires further clarification. Unfortunately, the clinical transplantation field currently relies on chronic non-specific immunosuppression to maintain graft survival and has not yet progressed to the point of using defined therapeutics to induce allograft tolerance in prospective trials. As such, it is challenging to determine the degree to which these or other potential pathways of tolerance impairment by immune memory pose significant barriers to achieving transplantation tolerance in humans.

**Table 1:** Characteristics of differing pathways whereby memory T cells impair allograft tolerance.

Pathway of tolerance blockade	Potential source of memory-directed antigen	Direct specificity for donor MHC molecules	Reactivity with donor versus host APCs	Potential clinical scenario	Pre-clinical evidence	Clinical evidence
1. Conventional heterologous immunity	Environment/pathogens/vaccination	Yes	Donor APCs and tissues	Recipient with common cellular immune memory	(8, 13–15, 69)	(12, 40, 67, 70–73)
2. Heterologous immunity from autoimmunity	Autoantigens	Yes	Donor *and* host APCs	Autoimmune recipient of organ transplant	(26)	Unknown
3. Linked recognition of donor-associated antigens	Pathogens or autoantigens	Not required	Host APCs	CMV+ or EBV+ organ transplanted into CMV+ EBV+ recipient	(43)	Unknown

## Author Contributions

AB and RG wrote and edited the review article. RG created the model figures and AB created **Table 1**. All authors contributed to the article and approved the submitted version.

## Funding

This work was supported by NIH grants RO1 DK115745 and UC4 DK104223 (RG) and by RO1 DK099187 (AB).

## Conflict of Interest

The authors declare that the research was conducted in the absence of any commercial or financial relationships that could be construed as a potential conflict of interest.
